# Proteins Recognizing DNA: Structural Uniqueness and Versatility of DNA-Binding Domains in Stem Cell Transcription Factors

**DOI:** 10.3390/genes8080192

**Published:** 2017-08-01

**Authors:** Dhanusha Yesudhas, Maria Batool, Muhammad Ayaz Anwar, Suresh Panneerselvam, Sangdun Choi

**Affiliations:** Department of Molecular Science and Technology, Ajou University, Suwon 443-749, Korea; dhanusha2504@gmail.com (D.Y.); mariabatool.28@gmail.com (M.B.); ayaz@ajou.ac.kr (M.A.A.); sureshcbt@gmail.com (S.P.)

**Keywords:** base and shape readouts, protein-DNA interaction, protein-DNA recognition, TF domain family

## Abstract

Proteins in the form of transcription factors (TFs) bind to specific DNA sites that regulate cell growth, differentiation, and cell development. The interactions between proteins and DNA are important toward maintaining and expressing genetic information. Without knowing TFs structures and DNA-binding properties, it is difficult to completely understand the mechanisms by which genetic information is transferred between DNA and proteins. The increasing availability of structural data on protein-DNA complexes and recognition mechanisms provides deeper insights into the nature of protein-DNA interactions and therefore, allows their manipulation. TFs utilize different mechanisms to recognize their cognate DNA (direct and indirect readouts). In this review, we focus on these recognition mechanisms as well as on the analysis of the DNA-binding domains of stem cell TFs, discussing the relative role of various amino acids toward facilitating such interactions. Unveiling such mechanisms will improve our understanding of the molecular pathways through which TFs are involved in repressing and activating gene expression.

## 1. Introduction

Most biological activities are governed by multiple protein-DNA interactions. The fundamental phenomenon underlying these interactions is the process by which proteins search and recognize their specific sites on the DNA, thereby enabling the transmission of genetic information to initiate various biological processes. Over the years, theoretical and experimental advances have allowed to improve our understanding of the mechanisms by which transcription factors (TFs) search for, and recognize these binding sites. In addition, researchers have explored how TFs interact with each other and with their binding partners. Although significant progress has been achieved toward understanding the TF search process, the details of this mechanism remain controversial [1,2].

One of the most puzzling phenomena involved in protein search over DNA is the effect of multiple targets, which is particularly important in eukaryotic genomes. Eukaryotic genomes harbor multiple target sites between tightly bound nucleosome core particles on accessible DNA fragments [1]. Recent studies showed that single nucleotide changes can alter TF selectivity, and also influence the sequence of events culminating in the TF binding with its true recognition site. Additionally, other major hurdles faced by TFs regarding their selectivity include the existence of cellular networks, dynamic protein-DNA conformational changes, and tight packing of multiple TFs at the regulator sites of a single DNA section [3]. These factors affect the complexity of protein-DNA recognition processes at both sequence and structural levels, meaning nucleotide sequences and their resulting 3D structures. Furthermore, other factors such as TFs’ flexibility for their binding sites, the influence of cofactors, cooperative binding of other TFs, DNA methylation, and other epigenetic modifications add to the complexity of this process. The effect of nucleosomes and their binding with TFs, chromatin accessibility, and nucleosome occupancy will also have an impact on TF-DNA readouts [4]. Along with the nucleosomes, the distribution of sequence-specific TFs (cell-specific and tissue-specific, but also ubiquitous) also greatly affects TF binding. Recent studies show that realistic observations about the readout mechanism vary across the various protein families [4,5,6]. Most of these readout mechanisms are discussed in this review.

Proteins use a wide range of DNA-binding structural motifs, such as homeodomain (HD), helix-turn-helix (HTH), and high-mobility group box (HMG) to recognize DNA. HTH is the most common binding motif and can be found in several repressor and activator proteins. Despite their structural diversity, these domains participate in a variety of functions that include acting as substrate interaction mediators, enzymes to operate DNA, and transcriptional regulators [7]. Several proteins also contain flexible segments outside the DNA-binding domain to facilitate specific and non-specific interactions. The phage Φ29 transcriptional regulator p4 uses its N-terminal beta-turn substructure for specific contact with DNA [8]. Likewise, HD proteins use N-terminal arms and a linker region to interact with DNA; for example, λ repressor uses its N-terminal arm to make contact with the major groove [9]. The Encyclopedia of DNA Elements (ENCODE) data suggest that about 99.8% of putative binding motifs of TFs are not bound by their respective TFs in the genome [10,11]. It is, therefore, clear that the presence of a single binding motif per TF is not adequate for TF binding.

Over the past decades, developments in computational and structural biology have offered an immense potential toward studying the protein-DNA recognition code. Crystal structures of protein-DNA complexes were first solved in the 1980s [12], and more than 1600 protein–DNA structures have since been deposited in the Protein Data Bank (PDB) [4]. This plethora of information has helped us to conclude that preferential binding of a TF to its cognate site is purely based on its physical interactions, for instance, the physical interaction between the amino acid side chain of the TF and the atoms of DNA base pairs [13]. Most of these physical interactions rely on hydrogen bonds, as well as on hydrophobic and water-mediated contacts. Other mechanisms driving protein-DNA interaction involve the recognition of DNA structural features by proteins; these structural features include the DNA major and minor grooves, backbone features, intrinsic curvature, hydration shells, as well as flexibility of DNA bending [14] and unwinding [15]. The dynamic behavior of DNA structure mostly governs the binding properties, and that can be understood through computational techniques [4,16]. Theoretical studies, such as molecular dynamics (MD) simulations, can provide additional information toward understanding protein-DNA complexes. The monitored dynamic movements of atoms reflect the functional and structural phenomena undergone by proteins or DNA during the initial phase of complex formation.

We have divided this review into two sections. The first section briefly discusses the DNA-recognition mechanisms, including historical mechanisms. The second section summarizes the major DNA-binding protein domains with reference to stem cell factors and their families. This section includes the structural properties of stem cell factor DNA-binding mechanisms and the cooperative binding phenomena driving target gene expression. Since stem cell factors are promising targets in the growing regenerative medicine field, researchers will benefit from the structural aspects of these factors provided in this review.

## 2. Binding Site Recognition and TFs

**Figure 1:**
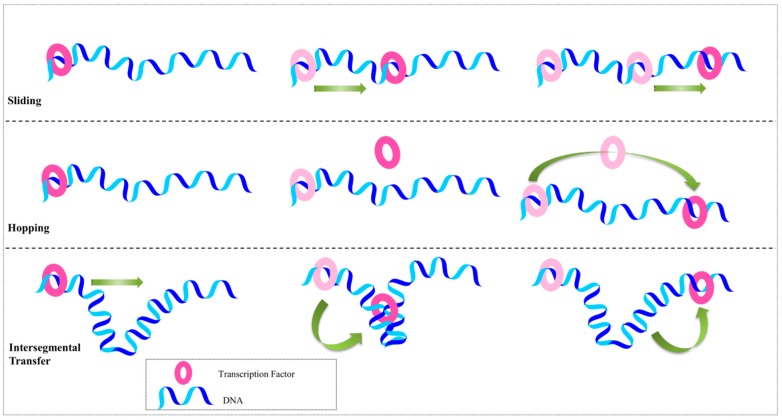
Protein-DNA recognition mechanisms. The main three protein-DNA recognition mechanisms are shown. When the transcription factor (pink ring) moves from one site to another by means of sliding along the DNA and is transferred from one base pair to another without dissociating from the DNA, this mechanism is called sliding (top). Hopping occurs when the transcription factor moves on the DNA by dissociating from one site and re-associating with another site (center). Intersegmental transfer describes the mechanism by which the transcription factor gets transferred through DNA bending or the formation of a DNA loop, resulting in the protein being bound transiently to both sides and subsequently moving from on site to the other (bottom).

Several mechanisms have been proposed to describe how TFs find their target sites on DNA. One of the main scenarios involves a ‘sliding’ mechanism, in which the protein moves from its initial non-specific site to its actual target site by sliding along the DNA (also known as 1-dimensional (1D) sliding) (Figure 1). The binding of the lactose (*lac*) repressor to non-operator sequences is an ideal example of sliding, since its DNA-binding entirely relies on electrostatic interactions, and consequently, diffusion occurs on an isopotential surface [17,18,19]. When the TF starts to move and shift counterions from the phosphate backbone, the same number of counterions binds to the site left free by the protein. The detailed sliding mechanism is explained later in this section. The sliding rate is also dependent on the hydrodynamic radius of the protein; the required rotational movement over the DNA backbone is greater for larger proteins, that tend to slide slowly [20,21]. Only a few DNA-binding proteins using the sliding mechanism from non-specific to specific binding have been structurally solved, among these, are BamHI [22], λ-repressor [23], and the lactose repressor [19,24].

The second scenario is a ‘hopping’ mechanism, in which a TF might hop from one site to another in 3D space by dissociating from its original site and subsequently binding to the new site. This may happen within the same chain and re-association occurs adjacent to the former dissociated site. For example, in the case of Herpes Simplex Virus Type 1 UL42, the binding between protein and DNA is mostly governed by electrostatic interactions, and its affinity is higher than that of the *lac* repressor [17]. Analyses indicated that non-electrostatic interactions play a key role toward allowing the binding of UL42, as well as facilitating its dissociation from the initial site. When condensation of counterions occurs at high salt concentrations, UL42 becomes more mobile and diffuses faster than at low salt concentrations [17]. Winter et al. [18] suggested that hopping should be slower than sliding, as its mechanism relies on dissociation followed by re-association to a different site. The hopping mechanism describes the search for DNA by proteins through the loss of electrostatic and non-electrostatic interactions, involving a microscopic dissociation constant (Figure 1).

A third search mechanism, proposed by Berg and von Hippel, is described as ‘intersegmental transfer’ [25]. In this scenario, the protein moves between two sites via an intermediate ‘loop’ formed by the DNA and subsequently bind at two different DNA sites (Figure 1). This mechanism is applicable to TFs with two DNA-binding sites (e.g., *Lac* repressor or SfiI endonuclease) [26]. Proteins with two DNA-binding sites can occasionally bind non-specifically to two locations situated far apart within the DNA strand, that are brought into close contact through the formation of these loops. Such TFs transfer across a point of close contact without dissociating from the DNA [27]. The EcoRV restriction enzyme binds to two specific or non-specific DNA sites in a deep cleft between two protein subunits, where the cleft is moderately narrow, in order to hold both duplexes simultaneously [28]. The search for a target site by a protein is accelerated through diffusion along the DNA strand [29]. This 3D diffusion can be assisted via the formation of a DNA loop allowing the protein to bind to two DNA segments simultaneously and thereby, enabling its transfer from one location of the segment to the other [30]. This intersegmental transfer accelerates the search for the DNA target site because this mechanism is based on constantly changing random configurations [25,31]. The impact of DNA conformation on target site searching is still unknown, but in the case of EcoRV restriction enzyme, the target finding rate is almost doubled when the DNA changes its conformation from a fully extended structure to a coiled structure [32]. Most of the searching mechanism studies are limited to naked DNA-protein complexes, which do not reflect the actual crowded environment of a cell. Studies have shown that many DNA-binding proteins travel a long distance by 1D diffusion. The search process for eukaryotes must occur in the presence of chromatin, which has the ability to hinder protein mobility. In this case, the protein must dissociate from the DNA, enter a 3D mode of diffusion state, and continue the target site searching process [33].

**Figure 2:**
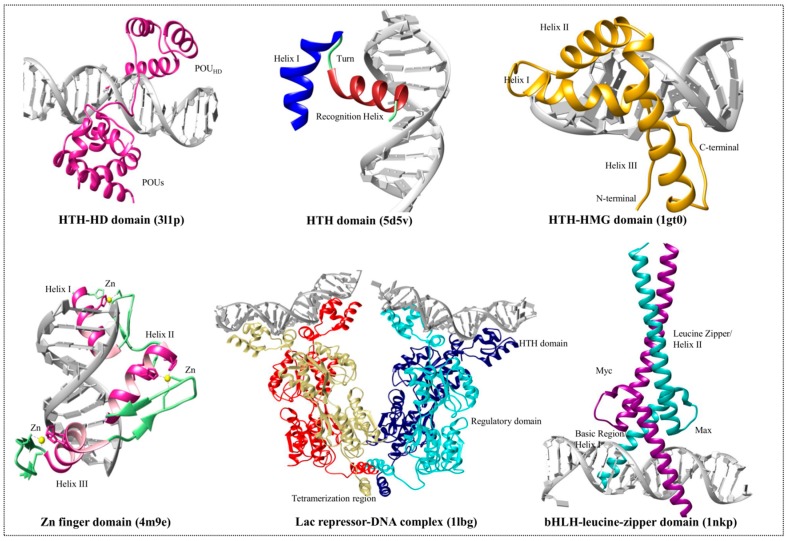
Representative figures of the transcription factor binding domains. The figure shows the crystal structures of different types of TF domains (3l1p, 4m9e, 5d5v, 1lbg, 1gt0, and 1nkp). The structures were obtained from the Protein Data Bank (PDB) and redrawn using chimera. The respective domains and important regions have been labeled. HTH stands for helix-turn-helix domain. bHLH stands for basic helix-loop-helix motif. HD and HMG stand for homeodomain and high-mobility group box domain, respectively.

The sliding and intersegmental transfer mechanisms can be explained through the example of the *lac* repressor. The *lac* repressor contains 4 identical monomers (a dimer of dimers) for its DNA-binding. The binding sequence of these dimers is symmetric or pseudo-symmetric, and each half is identified by these identical monomers [34,35,36]. The HTH domain of the *lac* repressor is the DNA-binding domain that facilitates the interaction with its target site on DNA (Figure 2). As a result of a rapid search (sliding) along the DNA molecule and intersegmental transfer between distant DNA sequences, the lactose repressor finds its target sites faster than the diffusion limit. The section comprised between residues 1–46 of the HTH domain, characterized by three α-helices, maintains its secondary structure through specific and non-specific binding. When the repressor binds to a non-specific site, the HTH domain interacts with the DNA backbone and maintains the interaction with its helix2 region in the major groove juxtaposition. This arrangement facilitates the interaction of helix2, the recognition helix, with the edges of the DNA bases, enabling the repressor to walk or search for its specific site on the DNA. The C-terminal residues of the DNA-binding domain, residues 47–62, form the hinge region, and are normally disordered during non-specific recognition; however, during specific site recognition, residues 50–58 acquire an α-helix configuration (hinge helix) [34]. The disordered hinge region and the flexibility of the HTH domain allow the protein to move freely along the DNA to search for its target site. In specific binding complexes, the hinge helix of each monomer is located at the symmetrical center of the binding site, thereby causing the hinge helices to interact with each other (intersegmental transfer) to allow better stability. Moreover, DNA bends at the symmetrical center of the specific binding site (37° angle), thereby supporting monomer-monomer interactions [24,35]. Experimental reports suggest that engineering a disulfide bond between the hinge helices of these monomers (Val52Cys mutation) will restrict the HTH domain movement of these monomers and thereby yield higher-affinity complexes [37].

### 2.1. Historical Mechanism: Base Readout vs. Shape Readout

The direct (physical interaction) and the indirect (alteration of the DNA shape) readout mechanisms are known as the historical mechanisms that drive protein-DNA interactions. Direct recognition occurs when the amino acid side chains of a protein interact with specific DNA bases [6]. Most protein-DNA interactions are mediated by direct physical interaction (hydrogen bonding or hydrophobic interactions) between the protein and the DNA base pairs. Specific binding is mainly obtained via hydrogen bonding between the protein and the major groove base pairs, because each of the four base pairs has a unique pattern of hydrogen bond donors and acceptors in the major groove [38]. The specificity of DNA-binding also depends on the number of hydrogen bonds existing between the protein and the major groove base pairs. Bidentate bonds (two hydrogen bonds with different donor and acceptor atoms) have a higher degree of specificity than bifurcated hydrogen bonds (two hydrogen bonds sharing the same donor). A normal single hydrogen bond does not contribute to specificity, whereas bidentate bonds do [13,39]. Hydrogen bonding between proteins and DNA is also facilitated by water molecules. Highly ordered water molecules mediate the specific base pair readouts in the major groove because they reflect the position of hydrogen bond donors and acceptors at the base edges. For example, the RXR/retinoic acid receptor (RAR)-DNA complex utilizes several lysine and arginine residues to mediate the specific readouts [40]. In the case of the *lac* repressor, the protein-DNA interface is enriched with water molecules when it binds non-specifically; however, this interface is devoid of water when it binds to specific sites [13,19].

Although the base readout exists in all protein-DNA complexes, the structure of bound DNA frequently deviates from its standard one. Often, these deviations also contribute to specific DNA-binding. For instance, papillomavirus E2 protein and the TATA box binding protein (TBP) both induce some degree of deformation in their cognate DNA to facilitate hydrogen bonding and non-polar interactions with the protein [41,42]. Such indirect methods for protein-DNA recognition are also known as shape readout (indirect readout) mechanisms, in which the binding relies on the base pairs that do not directly contact the protein, but instead, create structural changes within the DNA to facilitate recognition. The elusive conformational shift occurring in biomolecules is the transition among B, A, and Z conformations of DNA [43]. B-form DNA is the most favored conformation for a protein-DNA complex under physiological conditions, whereas A-form DNA is induced locally in some complexes under low water activity. The important factors driving this transition are hydration and electrostatics; however, solvent conditions, counterions condensation, and free energy contribution from phosphate-phosphate repulsion also contribute to this transition [44,45].

DNA bending and kinks are parameters contributing to the shape readout mechanism. DNA kinks result from the complete or partial loss of stacking energies at a single base pair step. Since kinks can occur at any individual base pair step, the adjacent region maintains its B-form conformation. Kinks and bending are stabilized upon protein binding, which compensates for the lack of base pair stacking energies. This energy compensation, resulting from the interaction of protein side-chain hydrophobic residues, makes the protein-DNA complex more stable [13]. Major/minor groove width also plays an important role in protein-DNA-binding. The differences in the hydrogen-bonding pattern of each base pair are due to different stacking energies and therefore, the stacking energies in each dinucleotide step affect the minor groove width [6]. Roll, helical twist, and propeller twist are the rotational parameters that determine the contraction of the minor groove. ApT base pair steps have negative roll angles, causing compression at the minor groove and favoring protein binding [46]. In A-DNA, ApT and ApA exhibit negative roll values and have bifurcated hydrogen bonds (A:T base pair) that lead to propeller twisting and enhance minor groove narrowing [47]. The minor groove of B-DNA shows more electronegative potential than the major groove because perfect B-DNA has a wide, shallow major groove and a narrow, deep minor groove. Likewise, the AT-rich DNA sequence in the minor groove exhibits greater electronegative potential than GC-rich sequences. Thereby, the AT-rich sequences at the minor groove attract the protein and create a bend/kink to enhance interactions. Therefore, high-affinity binding site sequences are more bendable at the minor groove than low-affinity sites, which are straight (sometimes bent into the major groove) and rigid [48]. Hence, DNA curvature and flexibility are also main parameters to be considered when determining the affinity of protein-DNA interactions.

### 2.2. Beyond the Recognition Mechanism

Other than the defined mechanisms, there are other factors that contribute to protein-DNA recognition. The majority of TFs possess an intrinsically disordered (ID) region, which is known to promote proteins’ recognition of specific binding sites. ID regions play important roles in the transition from non-specific to specific binding, and facilitate protein diffusion along the DNA [49]. These regions also control selectivity by forming specific interactions and folding into functional forms, similarly to globular proteins [50]. The tails and linear regions of IDs enhance specific binding affinity, as the charged tails provide constant electrostatic interactions with the available base pair atoms, thereby helping the protein to find and attach to its binding site [50,51]. As a result, the availability of free proteins and the rate of protein diffusion are reduced. The linker region in IDs bridges between multidomain proteins and increases the rate of intersegmental transfer [49,52]. Another interesting fact about ID regions is that without their 3D structural information, their functional sites can be predicted from their primary sequences [53,54]. Moreover, protein-protein interactions use ID regions by targeting small motifs [55]. Intramolecular contacts of ID segments often regulate DNA-binding affinity via a competitive binding mechanism. For example, the transcriptional activation of p53 depends on its interaction with a 70-kDa subunit of human replication protein A (hRPA70), which contains weak- and high-affinity DNA-binding domains [56]. These domains are connected by the intrinsically unstructured linker domain (IULD). The ID region increases the concentration of weak-affinity DNA-binding domains, which in turn, causes a barrier between p53 and hRPA70. The hRPA70 affinity for single-stranded DNA (ssDNA) is greater than that of p53; therefore, depending on the ID region length, flexibility, and orientation, the hRPA70 IULD regulates the binding of p53 to DNA [50]. Alternative splicing modulates DNA-binding by altering ID regions. Alternative splicing affects the number of disordered regions in the ID segments and inserts or deletes post-translational modification sites to improve protein-protein interactions. Finally, ID regions have also been proposed to amplify signals and mediate allosteric responses [50,57].

Cooperative binding is yet another facet of DNA recognition. For instance, HMG box 1 protein (HMGB1) participates in several processes by binding to DNA through two HMG boxes [58]. Cooperative binding prevails when the protein-protein interaction between adjacently bound TFs stabilizes the complex and enhances the transcription activity of both proteins. However, cooperative binding might alter the specificity by extending TF binding [59]. Yeast TFs (MATa2, MATa1, and Mcm1) regulate the genes responsible for mating. MATa2 functions to recruit other cofactors and repress gene expression; nonetheless, its DNA-binding specificity is driven by the cooperative binding of cell type-specific cofactors [60,61]. The haploid-specific genes are repressed by MATa2:MATa1 heterodimers, whereas MATa2:Mcm1 heterotetramers repress the mating-type a-specific genes. The recruitment of co-repressor proteins Tup1 and Ssn6 by MATa2 represses the gene expression of both complexes. In most cases, the cooperative interactions are perpetuated through direct physical contact, but the allosteric effects provoked by DNA structural deformation also contribute to the cooperativity [59,62].

During the search process, only the physical aspect of protein-DNA interaction is addressed, whereas the biological aspects (assisted diffusion, involvement of other TFs, etc.) are more complex and mostly unknown. Although the non-specific protein-DNA binding implies a weaker interaction, their binding mainly mediates the target protein activities. Usually, when the protein encounters DNA, it reaches a random non-specific location on the DNA by diffusion, and then, uses the intramolecular translation process (sliding, hopping, or intersegmental transfer) to search its specific position [63]. The final stage is governed by the formation of specific hydrogen and electrostatic interactions at the protein-DNA interface, resulting in a precise geometrical fit between the protein and its consensus DNA [64]. Therefore, binding to the target site may establish a free energy barrier between non-specific and specific binding conformations [65].

Hydrophobic interaction is the major energetic factor contributing to the protein-DNA complex formation, which is the outcome of bound water molecule release from non-polar surfaces. Ion pairs are also contributing to the protein-DNA complex thermostability through allosteric effects. To fully understand the kinetic and thermodynamic natures of protein-DNA complexes, the salt-independent part of the total electrostatic free energy should be physico-chemically interpreted [66]. Furthermore, to characterize the thermodynamic properties of a biomolecular complex, the changes in enthalpy and entropy have to be calculated among the equilibrium states of unbound and bound conformations. Moreover, single-base variation in the DNA sequence greatly impacts the equilibrium of protein-DNA complexes and consequently, alters thermodynamic properties. Therefore, this phenomenon should also be taken into account when conducting thermodynamic calculations [67].

## 3. DNA-Binding Domain Families

In this section, we discuss the major DNA-binding protein domains of stem cell factors (Oct4, Sox2, Nanog, c-Myc, and Klf4) and their related families. The major DNA-binding domains, homeodomains, are described using Oct4 and Nanog as examples, whereas high mobility group domains are explained with Sox2, a stem cell transcription factor. The helix-loop-helix (HLH) domain is explained taking c-Myc as an example and the zinc finger (ZF) domain is described with Klf4 protein. Apart from these stem cell TFs, several other proteins belong to main domain superfamilies’, and their DNA domain architectures (based on overall secondary structure contents) are summarized in Table 1.

### 3.1. Helix-Turn-Helix Motif

Several motifs combine to form a compact globular structure called a domain; therefore, a motif is believed to be incapable of folding and forming a stable structure, whereas a domain can. HTH is the simplest motif with two α-helices connected by a turn. The dimeric Arc repressor [68] and 3-helix bundle homeodomains [7] are examples of the simplest proteins with HTH motifs [69]. Generally, the HTH proteins dimerize, and each monomer identifies one side of a symmetric DNA sequence [70]. The HD and the HMG domain proteins fall under the same structural arrangement of DNA-binding motif and belong to the HTH superfamily. Both families have HTH in their DNA-binding motifs and exhibit similar behaviors. The recognition helix (mainly the second helix) of HTH motifs binds to DNA bases (especially at major groove) through hydrogen and hydrophobic interactions, whereas the other helices are involved in maintaining protein-DNA stability. Even though the HTH motifs are conserved, their orientation relative to DNA-binding and their structural context differ among different protein families [13]. Outside of the HTH motif, the remaining structures are distinct among all protein families. The representation of the domain families with the referenced crystal structures are shown in Figure 2.

### 3.2. High-Mobility Group Protein Families

The HMG box domain was initially recognized as a domain that mediates DNA-binding in chromatin-associated proteins of the HMGB type. Later, this domain was observed in other TFs and subunits of chromatin remodeling complexes. This domain can mediate non-sequence-specific (HMGB-type proteins) and sequence-specific (TF like Sox2) DNA-binding [71].

HMG proteins are abundant and ubiquitous nuclear proteins that bind to nucleosomes and induce chromatin structural changes. These non-histone proteins play important roles in transcription, replication, recombination, and DNA repair processes. Structural changes in chromatin are maintained by HMG superfamily proteins. Members of these families are highly expressed in eukaryotic cells; they acquire different structures and unique motifs to bind and affect chromatin fibers by transiently interacting with nucleosomes. HMG protein binding is highly dynamic, and is not confined to a particular site; mostly, these proteins associate in a ‘hit and run’ fashion [72]. HMG proteins regroup three superfamilies, HMGA, HMGB, and HMGN, characterized by the presence of acidic amino acid-rich C-termini. The domain structures of these HMG family proteins include either an AT hook (HMGA), a HMG-box domain (HMGB), or a HMG-nucleosomal binding domain (HMGN). However, each protein family has an exclusive functional motif that brings specific changes in its DNA-binding site and participates in distinct cellular functions [73]. Several reviews offer wide information on the structure and architectural functions of HMG family proteins [74,75,76,77].

HMGB proteins (HMG-Box1 and HMG-Box2) share over 82% sequence identity, and are most abundant in the nucleus and highly conserved [78]. The HMGB superfamily has a unique DNA-binding domain (HMG-box) composed of about 75 residues, which can bind to DNA with high affinity and cause structural deformations, such as bending, kinking, looping, and unwinding [79]. Every HMGB family protein contains two functional HMG box motifs and a highly acidic C-terminal end. The functional motif of HMGB is built by three α-helices folded into an L-shaped structure, and has the capacity to penetrate the DNA minor groove and sharply bend it. Minor differences between the HMG boxes confer specificity to different HMGB proteins, whereas the acidic tails modulate their affinity and contribute to nuclear localization. The protein-protein interactions exhibited by these HMGB domains are mediated by the C-termini (including the helix3 and tail region), but this region does not take part in DNA contact.

Sox2 is one of the HMGB (79 amino acid residues) TFs that binds to DNA in a sequence-specific manner [80]. The HMGB domain-containing proteins bind at the major groove. In contrast, the unique HMG domain of Sox2 binds at the minor groove of DNA and produces a bend (approximately 90°) in the DNA to improve affinity (Figure 2). Usually, if a protein carries more than one HMG domain (e.g., HMGB1, HMGB2, and upstream binding factor) it has a decreased DNA-binding specificity (non-specific binding), whereas the proteins with single HMG domain will bind in a sequence-specific manner (e.g., Sox proteins and T-cell factor/lymphoid enhancer factor family proteins) [81,82]. The sequence-specific Sox2-DNA interaction is mediated by numerous base pair-specific hydrogen bonds and is closely related to Sry/DNA interactions. Asn8, Ser31, Ser34, and Tyr72 are responsible for important protein-DNA interactions. Upon Sox2 binding, the side chain residues of helix1 and helix2 are inserted at the consensus sequence (CTTTGTT) and forces the DNA unwind and open. The C-terminal tail is unstructured in Sox proteins and is specific to these proteins, promoting interaction with DNA [83].

### 3.3. Homeodomain Proteins

The homeodomains are small, conserved domains with three helices containing approximately 60 amino acid residues, often found with additional flanking domains and cofactors. They have a common fold arrangement; helix1 and helix2 are antiparallel to each other while helix3 lies across both. In HD proteins, the third helix is considered to be the recognition helix and is responsible for interacting with DNA at the major groove. Based on 103 *Drosophila* homeobox genes, 16 and 11 major classes have been identified to date in animals and plants, respectively [84]. The sequence specificity of this domain is governed by the major groove interactions, and the minor groove contacts contribute to binding strength. Arginine, particularly at the 5th position of HD, is found in 99 of the 106 *Drosophila* HDs, and is preferentially found in narrow minor grooves [6]. Different HD domain families differ in their N-terminal arm regions [84], are rich in basic amino acids, and have a hydrophobic residue at the 8th position (start of helix1). In the helix1 region, the 16th and 20th positions are often occupied by hydrophobic residues. The amino acid residues Trp48, Phe49, Asn51, and Arg53 of helix3 are highly conserved within the HD region. The conservation of these residues promotes DNA-binding and overall stability [85,86].

HD domains bind to the genome in a context-dependent and cell/tissue-specific manner. They can drive lineage-specific transcription by recruiting the ubiquitous and tissue-specific TFs. For example, cone-rod homeobox (Crx), a retina-specific TF, recruits ubiquitous myocyte enhancer factor 2D (MEF2D) away from the canonical MEF2D binding site and redirects it to retina-specific enhancers [87]. The reprogramming activity of HD is observed in Oct4 [88] and Pax7 [89] proteins. HD proteins have the capacity to bind closed chromatin structures and enable co-activator binding to promote gene expression.

Although the HDs are conserved class-specific domains [84], they also possess 5–10 residues-long short linear motifs (SLiMs) that are involved in weak and transient interactions [90]. SLiMs are located within the long, disordered regions of the TFs, thereby playing an important role in interactions with a wide range of cofactors in a context-dependent manner [91]. However, the long and disordered regions pose a challenge for the specificity of HD proteins; the increasing number of contacts can displace the HD proteins from their binding sites. However, TFs with HD deletions and SLiM mutations show aberrant and allelic activities, explaining the importance of HD domains and their disordered region motifs.

**Figure 3:**
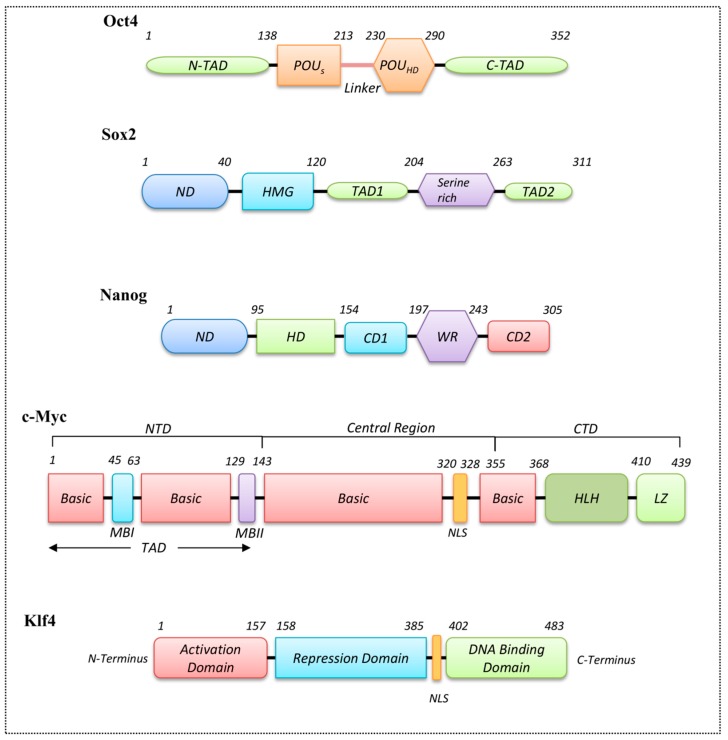
Domain architectures of stem cell transcription factors. A representation of the arrangement of functional domains in stem cell transcription factors Oct4, Sox2, Nanog, c-Myc, and Klf4 are shown. Each domain is marked with the length of its corresponding amino acid sequence. TAD stands for transactivation domain, HMG, HD, WR, HLH, and LZ stand for high-mobility group, homeodomain, tryptophan repeats, helix-loop-helix, and leucine zippers, respectively. NLS stands for nuclear localization sequence. MBI and MBII stand for Myc Boxes I and II, respectively. ND, CD1, CD2, and POU stand for N-terminal domain, C-terminal domain 1, C-terminal domain 2 and POU is derived from the names of three mammalian transcription factors, the pituitary-specific Pit-1, the octamer-binding proteins Oct-1 and Oct-2, and the neural Unc-86 from *Caenorhabditis elegans*.

The stem cell TF Nanog is another example of an HD domain protein, whose crystal structure has been published recently [92]. Nanog has a central HD domain composed of 60 amino acid residues, which is highly conserved in *Hox* genes, and preferentially binds at TAAT(G/T)(G/T). Along with the HD domain, Nanog (305 amino acids) possesses a serine-rich N-terminal domain (ND) and a C-terminal domain (CD) including the tryptophan repeats (WR) motif [93] (Figure 3). The HD domain has an unstructured N-terminus and three α-helices, with helix3 determining the binding specificity to the consensus sequence. Nanog’s HD domain is distinct from those observed in other HD proteins because it displays variant residues adopting non-canonical conformations during the interactions [93]. Helix1 and helix2 interact with the minor and major grooves, respectively, whereas helix3 interacts with the DNA backbone and helix1. Tyr119, Leu122, Gln124, and Lys137 are important residues for the Nanog-DNA interaction. Mutational studies showed that mutating residues Lys137, Thr141, Asn145, and Arg147 to alanine abolished the HD domain-DNA interaction [92].

Oct4, a POU family protein, is composed of two HTH DNA-binding domains known as POU-specific (POU_S_) and POU-homeodomain (POU_HD_) [94]. POU_S_ is only present in POU factors and is more conserved than the POU_HD_ domain, which is distantly related to the classic HD proteins. The POU_S_ domain has two short α-helices, which bind at the left half of the octamer motif through its HTH. The DNA-POU_S_ domain interaction is mediated by the third α-helix [95]. These bipartite subdomains (POU_S_ and POU_HD_) are connected by a linker region of variable length [95]. The unique variable linker region controls the reprogramming efficiency of Oct4, which has an α-helix (α5) structure [96], and helps to recruit other epigenetic factors (such as Sox2, Nanog, and others) to Oct4 for the reprogramming process [97]. The first five residues of the N-terminal arm of the POU_HD_ domain contain either lysine or arginine. This region fits into the minor groove and interacts with the 5′ end of its DNA-binding site [98]. The recognition helix of POU_HD_ is highly conserved with a unique cysteine residue at the 50th position, a characteristic that identifies the family members across the diverse phyla [85]. Although POU_HD_ domains are related to the classic HD domains, their DNA recognition mechanism is distinct and exhibits inefficient DNA-binding. Efficient DNA-binding is driven by the cooperative binding of POU_S_ and POU_HD_ at the major groove [97].

HD domain proteins prefer AT-rich regions; sites bound by HD TFs generally have very low GC content around the core binding sites [13]. Although a wealth of information is available regarding HD-DNA interactions, the exact mechanism remains elusive. HD-DNA recognition is thought to occur through the action of specific amino acids present in the HD recognition helix, which engage its corresponding nucleotides in the cis-regulatory elements. Other additional recognition mechanisms for HD domain proteins include water-mediated interactions, alterations in the DNA structural parameters, and the presence of cooperative binding factors [93].

### 3.4. Helix-Loop-Helix Proteins

The HLH family of TF comprises approximately 200 members; each member has a distinct function regarding cell cycle control and differentiation. The HLH domain mediates homo/hetero dimerization, thereby playing an important role in DNA-binding and transcriptional regulation [99]. Most HLH members contain highly basic residues close to the HLH domain that facilitate DNA-binding at the canonical E-box site (CANNTG). The highly conserved HLH region has two α-helices (15–20 residues-long), which are separated by a short loop of variable length [100].

A large number of HLH family proteins have been classified based on distribution, DNA-binding specificity, and dimerization capabilities [101]. Class I HLH proteins (E12, E47, HEB, E2-2, and Daughterless; also known as E proteins) are capable of forming homo- or heterodimers on the E-box binding site. The class II proteins (atonal, MyoD, myogenin, NeuroD/BETA2, and the achaete-scute complex) preferentially form heterodimers with the E proteins. Class III HLH proteins (Myc family of TFs, transcription factor binding to IGHM enhancer 3 (TFE3), sterol regulatory element-binding proteins (SREBP-1), and microphthalmia-associated TF (MITF)) [101,102] have leucine zippers (LZ) adjacent to the HTH domain motif. Class IV proteins (Mad, Myc-associated factor X (Max), and Max interacting protein (Mxi)) [103] dimerize with the Myc proteins. Members of this protein family that lack the DNA-binding region are known as Class V HLH proteins (inhibitor of DNA-binding proteins and emc (Extramachrochaetae) proteins) [104,105,106,107], which happen to function separately by dimerizing with other basic-HLH (bHLH) type TF and act as negative regulators toward the binding of bHLH proteins to DNA. Class V members are negative regulators of Class I and II HLH proteins as well [107]. Class VI and VII proteins contain proline in the basic region and bHLH-PER-ARNT-SIM domains, respectively [100,108,109].

The C-terminus of c-Myc harbors bHLH-zipper domain, and the amino-terminal end holds the two highly conserved elements (Myc box 1 and 2 (MBI and MBII)), which are necessary for the transactivation of its target genes. Mutations in the TAD domain or in the bHLH-zipper domain have the potential to abolish c-Myc activity [110]. The heterodimerization of bHLH proteins is mediated through two HLH-zipper interfaces, and for c-Myc, the heterodimerization occurs through a highly specific interaction with the bHLH-zipper protein Max. The heterodimerization of c-Myc with Max enables the association with E-box DNA sequences (CACGTG), thereby stimulating transcription (Figure 2) [111,112]. Max proteins homodimerize weakly, whereas forced Max expression blocks the c-Myc biological activity through competition for E-box sites [113]. Although many biological activities of c-Myc proteins are dependent on Max heterodimers, Max-independent functions of c-Myc proteins are also reported [114,115]. The c-Myc proteins are present with or without the Max protein at the non-E-box binding site with the help of other interacting TFs [115,116,117]. The c-Myc functional domains involved in transcriptional regulation are highlighted in Figure 3.

### 3.5. Zinc Finger Domain Proteins

The ZF is a large, widespread domain structure present in 3% of genes composing the human genome. A typical ZF domain contains two histidine and two cysteine residues that pack a zinc ion with coordinate bonds. This motif is composed of an α-helix and antiparallel β-strand (Figure 2); four key amino acid residues, located in specific positions at the tip of the finger, are responsible for DNA recognition by creating hydrogen bonds with the major groove [118]. Each ZF is tandemly linked in a polar fashion to recognize DNA of variable lengths [119]. Even though each finger domain has similar structural arrangements, variations in key amino acid residues drive a large number of combinatorial possibilities in terms of DNA sequence recognition. Thereby, the majority (approximately 80%) of the classical C2H2-ZF proteins have no known DNA-binding motifs [120,121]. Human ZF proteins have approximately 10 C2H2-ZF domains that can bind to approximately 30 base pairs on DNA; however, the entire domain does not necessarily bind simultaneously. Evidence suggests that this domain also binds to other proteins and ligands [122,123].

C2H2-ZF proteins can be divided into three major groups. The first group contains a cluster of three ZF, and is mainly composed of Sp1-like transcription factors [124]. TFs belonging to the Klf family (approximately 17 members in humans) have been identified, displaying three conserved ZF in their C-terminal polypeptide chains [125]. The second group is the smallest, with one or more pairs of ZF. The greater the number of ZF, the further they are located from each other. Tramtrack (TTK-one ZF pair), positive regulatory domain II binding factor (PRDII-BF1- two ZF pairs) [126], and basonudin (three ZF pairs) [127] are some examples of this group. The most abundant group is the third one, which comprises ZF proteins containing clusters made of four or more ZFs. Each protein may have one or more domains containing several closely spaced ZFs. TF CTCF (11 ZFs arranged in one cluster) [128,129], myeloid zinc finger 1 (MZF-1) [130], NEP1 –interacting protein (NIP1) [131], and zinc finger protein 394 (ZNF394) [132] are some examples. Although ZF proteins are classified into these three groups, there are other types of ZFs that contain both paired and clustered groups. For example, zinc finger protein 305 (ZNF305) and paternally-expressed gene 3 (PEG3) TFs contain both pairs and clusters composed of more than four ZFs [118].

The stem cell TF known as Klf4 has been characterized by the C2H2-ZF DNA-binding motif located at its C-terminus (Figure 3). The amino acid residue at position 81 in the ZF domain is highly conserved within the Klf family, and can recognize GC-rich regions (CACCC) [125]. The structural arrangement of the ZF domain of Klf4 comprises two short β-strands followed by an α-helix. The classical ZF arrangement displays a #-X-C-X(1-5)-C-X3-#-X5-#-X2-H-X(3-6)-[H/C] pattern, in which C, H, and X denote cysteine, histidine, and any amino acid, respectively. The # symbol marks an important amino acid, and the associated number defines the number of amino acid residues. Klf proteins have highly conserved linker residues (TGE(R/K)P(Y/F)X) between their ZF domains [125]. The N-termini of Klf proteins have other transcriptional regulatory domains that are specific to each Klf protein. This reflects the functional diversity of Klf proteins, resulting in various interactions with distinct co-activators and repressors [133]. A representation of the domain families with the referenced crystal structures and the domain organization of stem cell TFs are shown in Figure 2 and Figure 3, respectively.

## 4. Cooperative Binding of Stem Cell TFs

Oct4, Sox2, and Nanog are important TFs, essential toward maintaining the embryonic pluripotent state. Cooperative interactions between these factors drive the pluripotent-specific expression of target genes. Oct4 binds to DNA as a monomer, a homodimer, or a heterodimer with other transcription factors (e.g., Sox2). Even though the Sox family of TFs plays an extensive role in embryonic development, the binding of Sox2 alone does not initiate transcription; it requires a binding partner at an adjacent site on its targeted DNA. Even though Oct4/Sox2 play independent roles in determining other cell types, their cooperative interaction also drives the transcription of a specific set of target genes responsible for cell reprogramming. The known target genes of Oct4/Sox2 heterodimers include fibroblast growth factor 4 (*Fgf4*), undifferentiated embryonic cell transcription factor 1 (*Utf1*), F-box only protein 15 (*Fbxo15*), as well as *Sox2* and *Pou5f1* (the gene encoding Oct4) themselves [134].

**Figure 4:**
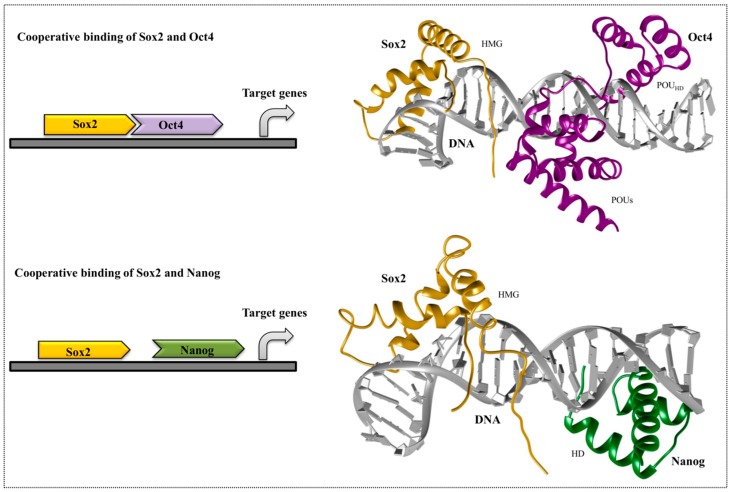
Representative figure of the cooperative binding of stem cell factors. The figure illustrates the cooperative binding of Sox2 and Oct4, as well as Sox2 and Nanog, on their enhancers/promoters of target genes. The Oct4/Sox2 crystal structure is obtained from PDB (1gt0), whereas Sox2/Nanog structure was modeled using chimera.

Proteins are generally dynamic in nature, and upon binding, protein complexes promote a state that is energetically favorable for efficient transcription. Oct4/Sox2 DNA recognition is driven by the sequence-dependent deformation of DNA, where Oct4, an HTH-containing protein, docks into the major groove of DNA to establish the direct sequence-specific interaction. This causes dynamic and transient contacts between the protein domains and the DNA-binding interface. Combined, the two domains (POU_S_ and POU_HD_) of Oct4 recognize the consensus sequence ATGC(A/T)AAT, where the POU_S_ recognizes the first half and the POU_HD_ recognizes the second [94,135]. Furthermore, the Sox2 HMG domain recognizes the CTTTGTT consensus sequence, and its binding introduces a topological deformation (pronounced kink) with respect to B-DNA. The residues of helix1 and helix2 of the Sox2-HMG domain are inserted at the 3-base pair stacks (which are marked) of the consensus sequence (C*T*T*TGTT), and unwind the DNA. Only helix1 and helix2 are needed to enable the DNA interaction, whereas the residues Pro68, Tyr72, and Pro74 from helix3 reorient the C-terminal tail and maintain the interaction with Oct4 [83]. The Oct4 residues Glu82 and Lys85 transiently create salt bridges with the complementary residues in Sox2 [97]. Mutating certain amino acids of the HMG residues of Sox factors correspondingly swaps their ability to dimerize with Oct4 on specific composite motifs and to direct cell fate decisions during the induction of pluripotency or endodermal fate (Figure 4).

Even though there is no structural information about the direct physical interaction among Oct4, Sox2, and Nanog, experimental evidence has shown that Nanog-bound promoters are often co-occupied by these two proteins. Within the *Nanog* promoter region, the presence of Sox-Oct-cis regulatory elements confirms the cooperative binding among these factors, which may be necessary for *Nanog* pluripotent transcription [134]. Various experiments have shown that Oct4 and Sox2 regulate their own transcription via Oct4/Sox2 heterodimerization, and positively regulate Nanog transcription to maintain an undifferentiated state in embryonic stem cells (ESCs). Direct physical interactions between Nanog and Sox2 can occur, in which the WR domain of Nanog and the transactivation domain of Sox2 interact (Figure 4). The cooperative binding between Nanog and Sox2 is mediated by the tryptophan residues located in the WR domain of Nanog, and residues 205–263 within the serine-rich region of Sox2. This critical Nanog-interacting region in Sox2 (residues 212–233) is highly enriched with hydroxyamino acids, similarly to the WR domain residues in Nanog [136] (Figure 3). Moreover, careful examination has highlighted three repeats of the sequence S X T/S Y of this 21-amino acid region that may be responsible for mediating the interaction with Nanog.

Studies have proven that Oct4, Sox2, and Klf4 can independently target the nucleosomes using partial or degenerate motifs, and can also recognize the full canonical motifs in the absence of nucleosomes [137]. This differential ability of TFs to recognize their target sites on nucleosomes paves a way to build a hierarchical model for the pioneer factors and identify their targets in silent chromatin structures [138].

The gene regulatory functions are carried out through the molecular interactions between protein and DNA. Uncovering such interactions, as well as identifying precise genome-wide DNA-binding sites for TFs, is performed by chromatin immunoprecipitation, using the DNA sequencing (ChIP-Seq) method [139]. A comprehensive map of Myc binding has been identified within the mouse ESCs genome through ChIP-Seq method. This map revealed 4325 Myc binding sites, of which 2885 were newly identified [140]. Hundreds of target genes have been identified for the Oct4/Sox2 binding, in which Zfp206 is an important TF, playing a role in maintaining stem cell pluripotency [141]. Along with this typical ChIP assay, recent computational methods are also gaining importance toward genome-wide studies on protein-DNA interactions. Position weight matrix (PWM) is the method used for discovering motifs in nucleotide or amino acid sequences, which incorporates the most advanced algorithm in bioinformatics [142]. This method has been extensively used in finding the transcription initiation sites, intron splicing sites, whole-genome screening of regulatory elements, and is also a useful measure for motif strength [143,144,145].

## 5. Summary and Perspective

In this review, we summarized the recognition mechanisms that TFs utilize to select their DNA-binding sites. Base readout involves hydrogen bonds and hydrophobic interactions between the DNA bases and the protein, enabling direct readouts. The shape readout mechanism is utilized by many TFs, where the normal or deformed shape of DNA attracts its perfect binding partner. DNA bending, unwinding, and other helical parameters, like major/minor groove width, also influence the shape readout mechanism. The initial search scenarios such as sliding, hopping, and intersegmental jumping are also discussed. To study the structural details and their cooperative mechanisms, we also discussed TF binding domain families with a special emphasis on the stem cell TFs Oct4, Sox2, Nanog, c-Myc, and Klf4. Specifically, the DNA-binding domains of HTH, HLH, and ZF domain structures, as well as their recognition mechanisms, were discussed. Taken together, this review provides basic and advanced information about the recognition mechanisms of TFs, along with a structural study of stem cell factors.

A widely used method to identify the binding site for a protein is PWM [142]. The interdependencies between the nucleotide positions in the binding site can justify quantitative binding, whereas the qualitative binding relies on cofactors, cooperativity, and chromatin accessibility. An accurate model to represent all these highly complex, dependent attributes in protein-DNA binding remains elusive; however, these questions can be resolved at a molecular level by MD simulation methods. These may include the impact of superhelicity on protein-DNA interactions and thorough energy landscape analysis for protein-DNA recognition mechanisms [146]. Experiments have suggested that regulatory proteins should bind firmly to their target sites; however, they also found strong binding to other non-specific sites that act as bait, thereby increasing the time needed for a TF to discover its actual target site [147]. Numerous theoretical models and mechanisms have been proposed to solve this problem, but the predictions of these developing computational models have yet to be tested. Even though the single-molecule technique receives considerable attention regarding the examination of the motion of protein on DNA, it often lacks sufficient resolution to distinguish between sliding and hopping mechanisms [31,148,149]. However, the emerging super-resolution microscopy methods are capable of making direct visualization of individual proteins, which is useful to study DNA repair events [150,151]. Beyond this method, photoactivated localization microscopy (PALM) is used to track the movement of individual molecules in the living cells, which is very useful to study search mechanisms of protein-DNA complexes. In addition to that, PALM provides a direct way of counting the molecules in the cells, and provides a quantitative description of complex reaction networks [150,152]. Advancements in computational processing (graphics processing unit, GPU) and the optimization of MD algorithms have allowed us to analyze conformational ensembles, which represent the real macromolecules in a superior manner. Tools exist in MD simulations that make the setup of a macromolecular system much easier, taking into account macromolecules’ flexibility and the dynamic properties (especially thermodynamics). More recent computational methods use machine learning methods to find the characteristic features of protein-DNA interactions. With the availability of recent research contributions and with improved computational techniques, a number of strategies have been utilized toward determining protein-DNA interaction mechanisms. These approaches can be highly successful when combined with experimental data.

## Figures and Tables

**Table 1:** Different domain family proteins and their domain architectures.

No	Superfamily Proteins	Domain Motifs	Architecture of DNA-Binding Domains	Representative PROTEIN
1	Winged HTH proteins	Helix-turn-helix	mainly α	hRFX1
2	GCM domain	β-sheet	mixed α/β	WRKY transcription factor
3	Zinc-coordinating proteins	Zinc finger	mixed α/β	SIP1, FOG, Msn2p, A20, Klf4
4	ββα Zinc-finger family	Zinc finger	mixed α/β	Egr-1
5	Loop-sheet-helix family	Helix-turn-helix	mainly α	p53
6	Leucine zipper family	Helix-loop-helix	mainly α	Jun, Fos
7	POU domain	Helix-turn-helix	mainly α	Oct1, Oct2, Oct4
8	Copper-fist	Zinc finger	mixed α/β	Mac1
9	Histone-fold	NA	mainly α	TBP, TAF proteins, HuCHRAC
10	ETS domain	Helix-turn-helix	mainly α	pointed-P2
11	Bet v1-like	NA	mixed α/β	VASt
12	P-loop domain	NA	multidomain, mixed α/β	ARTS
13	TEA domain	NA	NA	Simian virus 40 (SV40), enhancer factor TEF-1
14	LytTR domain	NA	NA	AlgR/AgrA/LytR family of transcription factors
15	Steroid receptor	Zinc finger	mixed α/β	NA
16	p53-like transcription factors, E-set domains, and Runt domain proteins	Immunoglobulin-like β-sandwich motif	mainly β	NF-κB and Rel
17	TATA-box binding protein-like	TBP (TATA-binding protein) β-sheet	mainly β	HMGB1, HMGB2
18	DNA/RNA polymerases	NA	multidomain, mixed α/β	RNA polymerase I, II, III, IV and V
19	Ribbon-helix-helix	Ribbon-helix-helix	mixed α/β	CopG, NikR, ParG
20	HMG-box	Helix-turn-helix	mainly α	TCF-1, SRY
21	IHF-like DNA-binding proteins	NA	mixed α/β	HBsu
22	RNase A-like	NA	mixed α/β	Train A
23	TrpR-like	Helix-turn-helix	mainly α	TrpR like proteins
24	T4 endonuclease V	Helix-turn-helix	mainly α	RuvC protein
25	ARID-like	Helix-turn-helix	mainly α	SWI-SNF complex protein p270

The superfamily proteins were taken from the structural classification of proteins (SCOP) database and the information was retrieved and updated from Rohs’s work [13]. NA: Not available.
